# lncRNA RP11-34D15.2 sponges miR-223 to promote the PGC-1α/irisin signaling pathway, contributing to increased FFA and insulin resistance in obese children

**DOI:** 10.1530/EC-25-0028

**Published:** 2025-06-10

**Authors:** Shuang Guo, Mengnan Lu, Yuesheng Liu, Hongai Zhang, Biyao Lian, Yanfeng Xiao, Chunyan Yin

**Affiliations:** ^1^ Department of Pediatrics, Second Affiliated Hospital of Xi’an Jiaotong University, Xi’an, Shaanxi, China; ^2^ Department of Neonatology, Shanghai General Hospital Affiliated To Shanghai Jiao Tong University School of Medicine, Shanghai, China

**Keywords:** insulin resistance, lncRNA RP11-34D15.2, miR-223, free fatty acids, pediatric obesity

## Abstract

**Background:**

The global surge in pediatric obesity is closely linked to insulin resistance (IR) and type 2 diabetes, where adipose tissue free fatty acid (FFA) overload and mitochondrial dysfunction play pivotal roles. Long non-coding RNAs (lncRNAs) are emerging regulators of metabolic diseases, but their mechanistic contributions to childhood obesity-associated IR remain underexplored.

**Objective:**

This study investigates whether lncRNA RP11-34D15.2 modulates FFA-induced IR through the miR-223/PGC-1α/irisin signaling axis in obese children.

**Methods:**

We analyzed serum FFA, insulin, irisin, and white adipose tissue (WAT) transcriptomes in 40 obese and 40 normal-weight children. Functional validation included dual-luciferase reporter assays, primary adipocyte models, and high-fat diet (HFD) mice treated with lncRNA-specific shRNA (*n* = 10 per group). Molecular interactions were verified via RNA immunoprecipitation and western blotting.

**Results:**

Obese children exhibited 2.1-fold higher FFA levels and HOMA-IR (*P* < 0.01), but 38% lower serum irisin compared to controls, with irisin inversely correlating with body fat percentage (*r* = −0.67, *P* = 0.003). lncRNA RP11-34D15.2 was downregulated by 4.3-fold in obese WAT and positively correlated with irisin expression (*r* = 0.603, *P* = 0.018). Mechanistic studies revealed that lncRNA directly binds miR-223 (RIP-seq fold enrichment = 5.2, *P* = 0.004), relieving miR-223-mediated suppression of PGC-1α. Overexpressing lncRNA in adipocytes increased PGC-1α (2.8-fold) and irisin (1.9-fold), upregulated mitochondrial genes (CPT-1: 3.1-fold; UCP-1: 2.4-fold, *P* < 0.01), and reduced extracellular FFA by 44%. In HFD mice, lncRNA knockdown exacerbated glucose intolerance (AUC increased 29%, *P* = 0.007), whereas irisin supplementation restored insulin sensitivity (*P* = 0.013).

**Conclusion:**

lncRNA RP11-34D15.2 functions as a ceRNA sponging miR-223 to activate PGC-1α/irisin-mediated mitochondrial β-oxidation and FFA clearance, identifying therapeutic targets for childhood obesity.

## Introduction

Over the past three decades, the global prevalence of childhood obesity has experienced a dramatic surge, accompanied by a concomitant rise in the incidence of insulin resistance (IR) and type 2 diabetes mellitus (T2DM) (1). IR represents a critical transitional phase in T2DM pathogenesis, with pediatric patients progressing from IR to overt diabetes and associated complications at an accelerated rate compared to adults (2). Recent studies highlight regional disparities, with low- and middle-income countries experiencing accelerated rates of pediatric obesity due to urbanization and dietary shifts (3). Once pancreatic β-cell dysfunction develops, therapeutic options become largely restricted to glycemic management, often resulting in inadequate clinical outcomes and an elevated risk of diabetes-related complications (3).

Elevated circulating free fatty acids (FFAs) – observed in 20–30% of obese individuals compared to normal-weight counterparts – are well-established contributors to IR in key metabolic tissues, including skeletal muscle, liver, and adipose tissue (4). Mechanistically, FFAs activate toll-like receptor 4 (TLR4) signaling in adipocytes, triggering pro-inflammatory cytokine release and impairing insulin receptor substrate 1 (IRS-1) phosphorylation (5, 6). It is understood that saturated fatty acids such as palmitic acid can, for instance, inhibit KLF4 expression and activate the TLR4/NF-κB signaling pathway, promoting the expression of factors such as galectin-3 and inducing macrophage inflammation and IR, which involves increased NF-κB phosphorylation and the release of pro-inflammatory cytokines such as TNF-α and IL-6, ultimately leading to the inhibition of IRS-1 phosphorylation (7). Obesity-associated hyperlipidemia stems from both enhanced lipolysis and impaired FFA clearance (8). Adipose tissue regulates FFA metabolism primarily through mitochondrial β-oxidation and thermogenic pathways. Notably, mitochondrial dysfunction in adipocytes of obese children disrupts FFA catabolism, leading to systemic FFA accumulation and consequent IR (9). Dysfunctional mitochondria exhibit reduced oxidative capacity due to downregulation of electron transport chain complexes, as evidenced by recent studies in pediatric adipose tissue (10).

Chronic low-grade inflammation is increasingly recognized as a key driver of adipose mitochondrial dysfunction. However, recent studies emphasize the critical role of adipokines in maintaining mitochondrial homeostasis and FFA metabolism. Irisin, a PGC-1α-dependent myokine and adipokine encoded by the FNDC5 gene, has emerged as a central regulator of mitochondrial energy expenditure (11, 12). Circulating irisin levels are reduced in obesity and correlate more strongly with its adipose tissue expression than with skeletal muscle, highlighting its tissue-specific metabolic role (13). Recent studies further associate irisin deficiency with accelerated β-cell failure in adolescents with prediabetes (14), a condition potentially exacerbated by the detrimental effects of FFAs, which are known to promote inflammation via pathways such as TLR4 activation in adipose tissue macrophages, induce mitochondrial dysfunction through oxidative stress, and cause lipotoxicity via endoplasmic reticulum stress and JNK pathway activation, all of which collectively impair insulin signaling and can contribute to β-cell stress and dysfunction (15). This suggests that impaired irisin signaling further exacerbates mitochondrial inefficiency, perpetuating FFA accumulation and IR.

Long non-coding RNAs (lncRNAs) – defined as non-protein-coding transcripts exceeding 200 nucleotides – play diverse roles in epigenetic regulation, chromatin remodeling, and post-transcriptional gene modulation (16). These molecules interact with DNA, RNA, and protein complexes to influence genomic imprinting, cellular trafficking, and metabolic homeostasis (17). Notably, lncRNAs contribute to T2DM pathogenesis by modulating hepatic insulin signaling and lipid metabolism, often functioning as competing endogenous RNAs (ceRNAs) that sequester microRNAs (miRNAs) (18, 19, 20). For instance, lncRNA MEG3 modulates hepatic gluconeogenesis by sponging miR-214 (21), or – as observed in obesity where Meg3 expression is downregulated – by impairing glucose homeostasis and insulin signaling through mechanisms involving the induction of cellular senescence in hepatic endothelium and alterations in Akt phosphorylation and FoxO1 activity, thereby affecting hepatic glucose output and exacerbating liver inflammation (22), while lncRNA GAS5 has been shown to improve insulin sensitivity by acting as a competitive endogenous RNA for miR-28a-5p, which in turn prevents the inhibition of MARCH7, an E3 ubiquitin ligase, leading to the ubiquitination and degradation of the NLRP3 inflammasome. This suppression of NLRP3-mediated pyroptosis reduces the release of IL-1β and IL-18, thereby ameliorating adipose tissue inflammation and enhancing insulin sensitivity through the IRS-1/Akt pathway in models of metabolic disease (23). Despite these insights, the specific mechanisms by which lncRNAs contribute to pediatric obesity-associated IR remain poorly understood.

This study investigates lncRNA RP11-34D15.2, elucidating its role in IR development through miR-223 sponging and subsequent disruption of PGC-1α-mediated pathways in obese children. Our findings provide novel mechanistic insights into the interplay between lncRNAs, adipokine signaling, and metabolic dysregulation in pediatric obesity.

## Materials and methods

### Study participants

A prospective cohort of 40 obese and 40 normal-weight Chinese children aged 7–14 years undergoing elective noninfective surgical procedures (primarily cryptorchidism and hernia repair) was consecutively enrolled from the Pediatric Surgery Department of the Second Affiliated Hospital of Xi’an Jiaotong University between December 2018 and January 2020. Obesity classification followed the *Overweight and Obesity Screening Criteria for School-Aged Children and Adolescents* (20), with diagnostic thresholds including body mass index (BMI) ≥95th percentile for age/sex, waist circumference (WC) >90 cm (boys), or >80 cm (girls). Exclusion criteria comprised: i) genetic obesity syndromes, ii) infectious or autoimmune diseases, iii) malignancies, iv) recent (within 3 months) use of antibiotics, immunomodulators, or herbal medications, v) endocrine disorders including hormonal deficiencies, vi) malnutrition, vii) established type 2 diabetes mellitus (T2DM), and viii) medications affecting blood pressure, glucose, or lipid metabolism. The Institutional Review Board of Xi’an Jiaotong University approved the study protocol (approval ID: 2018254), with written informed consent obtained from all legal guardians.

### Anthropometric and biochemical assessments

Standardized anthropometric measurements included: body weight (kg), height (cm), WC (cm), hip circumference (cm), systolic blood pressure (SBP, mmHg), and diastolic blood pressure (DBP, mmHg). Age-, sex-, and height-adjusted standard deviation scores (SDS) for BMI (SDS-BMI), SBP (SDS-SBP), and DBP (SDS-DBP) were calculated using the LMS (lambda-mu-sigma) method (24), referencing normative data from Chinese pediatric populations. Fasting venous blood samples were collected for comprehensive biochemical profiling: plasma glucose (mmol/L), total cholesterol (TC, mmol/L), triglycerides (TG, mmol/L), low-density lipoprotein (LDL, mmol/L), and high-density lipoprotein (HDL, mmol/L) were quantified using standardized enzymatic assays. Serum FFAs (μmol/L) and irisin (ng/mL) concentrations were determined via commercial enzyme-linked immunosorbent assays (ELISA; Excell Biotech, China), while insulin levels (μIU/mL) were measured by radioimmunoassay (BeiFang Systems, China). Subcutaneous adipose tissue specimens obtained during surgical procedures were immediately snap-frozen in liquid nitrogen for subsequent molecular analyses.

### Calculations

The homeostatic model assessment index for insulin resistance (HOMA-IR) was evaluated using the following formula:

HOMA-IR = FPG (mmol/L) × FINS (mIU/L)/22.5

The anthropometric indices, such as SDS-SBP, SDS-DBP, and SDS-BMI, were calculated using the following formulas:

BMI was calculated according to the equation: BMI = body weight (Kg)/height^2^ (m^2^). As BMI and BP change with age and sex, BMI standard deviation score (BMI-SDS), and SBP and DBP SDS were calculated by the LMS method (24) for each one, according to the references of BMI and BP distribution of Chinese children and adolescents:

SDS-BMI = [BMI/M]L − 1/LS

SDS-SBP = [SBP/M]L − 1/LS

SDS-DBP = [DBP/M]L − 1/LS

### lncRNA microarray analysis

Total RNA was isolated from subcutaneous adipose tissue of four randomly selected obese and four normal-weight children using TRIzol® reagent (Invitrogen, USA). RNA purity (A260/A280 ratio) and integrity (RNA integrity number, RIN) were verified using an Agilent 2100 Bioanalyzer (Agilent Technologies, USA). Comprehensive lncRNA profiling was performed using the Arraystar Human LncRNA Array v4.0 platform (Arraystar Inc., USA). Following cDNA labeling and hybridization, arrays were washed and scanned using an Agilent G2505C microarray scanner. Differentially expressed lncRNAs were identified with stringent thresholds: fold change >2.0 and adjusted *P*-value <0.05.

### Cell culture and transfection

SW872 preadipocytes (ATCC® HTB-92™) were cultured in Dulbecco’s modified Eagle’s medium (DMEM) supplemented with 10% fetal bovine serum (Gibco, Thermo Fisher Scientific, USA) at 37°C in a humidified 5% CO_2_ atmosphere. Adipogenic differentiation was induced using a standard cocktail containing 0.5 mM 3-isobutyl-1-methylxanthine (IBMX), 1 μM dexamethasone, and 10 μg/mL insulin. At 70% confluence, cells were transfected with:

- adenoviral expression vectors encoding wild-type lncRNA RP11-34D15.2 (pAd-RP11-34D15.2),
- mutant lncRNA RP11-34D15.2 with deleted miR-223 binding sites (pAd-RP11-34D15.2-MUT),
- RP11-34D15.2-specific small interfering RNA (siRNA; GenePharma, China),
- using Lipofectamine™ 3000 transfection reagent (Thermo Fisher Scientific). Cells were harvested 48 h post-transfection for downstream analyses.

### RNA extraction and qRT-PCR

Total RNA was extracted from adipose tissue or SW872 cells using TRIzol® reagent (Takara Bio, China). Reverse transcription was performed using PrimeScript™ RT Master Mix (Takara), followed by quantitative PCR on an ABI StepOnePlus™ Real-Time PCR System (Applied Biosystems, USA). SYBR Green Master Mix (Bio-Rad, USA) and TaqMan™ miRNA assays (Thermo Fisher Scientific) were employed for mRNA and miRNA detection, respectively. Custom primers for lncRNA RP11-34D15.2, miR-223-5p, FNDC5/irisin, and PGC-1α were designed and synthesized by Sangon Biotech (China). β-actin and U6 small nuclear RNA served as endogenous controls for mRNA and miRNA normalization, respectively. Relative gene expression was calculated using the comparative 2^−ΔΔCt^ method.

### Western blotting

Total protein was extracted from differentiated SW872 adipocytes using RIPA lysis buffer (Beyotime Biotechnology, China) supplemented with protease inhibitors. Protein samples (30 μg per lane) were separated by 10% SDS-PAGE and transferred to polyvinylidene difluoride (PVDF) membranes (Millipore, Germany). Membranes were probed overnight at 4°C with the following primary antibodies: anti-FNDC5/irisin (1:200 dilution, ab174833; Abcam, UK), anti-carnitine palmitoyltransferase 1 (CPT-1, 1:300, ab234111), anti-uncoupling protein 1 (UCP-1, 1:300, ab10983), and anti-PGC-1α (1:500, ab54481; all from Abcam). After incubation with horseradish peroxidase-conjugated secondary antibodies (1:3,000; BosterBio, China), protein bands were visualized using an enhanced chemiluminescence (ECL) detection system (Bio-Rad). Densitometric analysis was performed using ImageLab™ software (Bio-Rad).

### Luciferase reporter assay

Wild-type (WT) and mutant (MUT) sequences of lncRNA RP11-34D15.2 and the 3′-untranslated region (3′-UTR) of PGC-1α containing predicted miR-223-5p binding sites were cloned into pmirGLO dual-luciferase reporter vectors (Promega, USA). HEK293 cells were co-transfected with either WT or MUT reporter constructs and miR-223-5p mimics (or negative control) using Lipofectamine® 2000 transfection reagent (Invitrogen). After 48 h, luciferase activity was quantified using the Dual-Glo® Luciferase Assay System (Promega), with firefly luciferase signals normalized to Renilla luciferase activity for data analysis.

### *In vivo* experiments

Twenty male C57BL/6J mice (8 weeks old) were fed a high-fat diet (HFD; 60% kcal from fat) for 16 weeks. Mice were randomized into two groups (*n* = 10 per group) with comparable baseline body weights (control: 38.7 ± 3.4 g vs lnc-RP11-34D15.2-shRNA: 37.6 ± 2.8 g; *P* > 0.05): i) lnc-shRNA control (tail vein-injected with scrambled shRNA plasmid) and ii) lnc-RP11-34D15.2-shRNA (injected with RP11-34D15.2-specific shRNA plasmid). Injections were administered twice weekly for 2 weeks. Post-euthanasia via CO_2_ inhalation, perirenal adipose tissue (PAT) was excised and weighed. Serum FFA, insulin, and irisin levels were quantified as above. Adipose tissue mRNA and protein levels of target molecules were assessed via qRT-PCR and western blotting.

### Statistical analysis

Data are expressed as mean ± standard deviation (SD). Normality was assessed via Shapiro–Wilk test; non-normal data were log-transformed. Intergroup comparisons used Student’s *t*-test or one-way ANOVA with Tukey’s post hoc test. Correlations were evaluated via Pearson’s coefficient. Analyses were performed using SPSS 22.0 (IBM Corp., USA), with *P* < 0.05 considered statistically significant.

## Results

### FNDC5/irisin is significantly reduced in obese children and negatively correlates with FFA and HOMA-IR

Comparative analysis of anthropometric and metabolic parameters between 40 obese and 40 normal-weight children revealed no significant intergroup differences in gender, pubertal stage, or age. However, obese children exhibited significantly elevated BMI, SDS-BMI, weight, WC, and waist-hip ratio (WHR) compared to controls (Table 1). Metabolic profiling demonstrated markedly higher serum FFA, insulin, and HOMA-IR levels in the obese cohort (*P* < 0.05; Table 2). Circulating irisin concentrations in obese children were approximately 30% lower than in normal-weight counterparts and displayed significant negative correlations with SDS-BMI, WHR, waist-to-height ratio (WHtR), and WC (*P* < 0.05). Notably, irisin levels remained inversely associated with insulin, LnHOMA-IR, and FFA even after adjustment for SDS-BMI and age.

**Table 1:** Comparison of anthropometric indexes, metabolic indexes, and irisin levels between the two groups of children.

Characteristic	normal-weight (40)	obese (40)	*P*
Age (y)	9.40 ± 1.82	10.70 ± 2.2	0.067
Sex (*n*)	male (23)/female (17)	male (21)/female (19)	0.054
BMI (kg/m^2^)	18.53 ± 1.73	26.79 ± 3.57^†^	0.002
SDS-BMI	2.46 ± 0.43	2.97 ± 0.45^†^	0.003
Weight (kg)	41.47 ± 8.95	58.54 ± 10.63^†^	0.002
WHtR	0.47 ± 0.32	0.65 ± 0.21	0.054
WC (cm)	63.35 ± 7.16	90.57 ± 11.23^†^	0.000
WHR	0.83 ± 0.05	0.92 ± 0.07^†^	0.001
SBP (mmHg)	97.30 ± 4.90	106.58 ± 12.70^†^	0.000
DBP (mmHg)	74.30 ± 2.50	78.00 ± 3.80*	0.034
FPG (mmol/L)	4.49 ± 0.63	5.25 ± 0.57	0.076
HOMA-IR	2.63 (1.57, 4.36)	3.64 (1.98, 5.26)*	0.043
TC (mmol/L)	3.57 ± 0.72	3.82 ± 0.63	0.073
TG (mmol/L)	0.87 ± 0.31	1.32 ± 0.49*	0.031
HDL-C (mmol/L)	1.29 ± 0.41	1.42 ± 0.39	0.058
LDL-C (mmol/L)	2.56 ± 0.93	2.98 ± 1.05*	0.029
FFA (umol/L)	208.75 ± 30.16	287.59 ± 41.56^†^	0.002
Irisin (ng/mL)	312.75 ± 43.37	185.54 ± 32.28^†^	0.001

Abbreviations: BMI, body mass index; SDS-BMI, BMI s.d. score; WHtR, waist-height ratio; WC, waist circumference; WHR, waist-to-hip ratio; SBP, systolic blood pressure; DBP, diastolic blood pressure; SDS-SBP, SBP s.d. score; SDS-DBP, DBP s.d. score; FPG, fasting plasma glucose; HDL-C, high-density lipoprotein cholesterol; HOMA-IR, homeostasis model of insulin resistance; LDL-C, low-density lipoprotein; TC, total cholesterol; TG, triglycerides; Data are expressed as mean ± s.d. or median (25th percentile, 75th percentile).

* *P* < 0.05.

^†^ *P* < 0.01 compared with obese.

**Table 2:** Correlation analysis between irisin and anthropometric and metabolic indexes of obese children.

Parameter	*r* *	*P* *	*r* ^†^	*P* ^†^
**Anthropometric parameters**				
BMI (kg/m^2^)	−0.535	<0.05		
SDS-BMI	−0.603	<0.05		
WC (cm)	−0.591	<0.05		
WHR	−0.587	<0.05		
WHtR	−0.564	<0.05		
**Metabolic parameters**				
FPG (mmol/L)	−0.342	0.021	−0.192	0.073
Insulin (pmol/L)	−0.475	<0.05	−0.254	<0.05
Ln HOMA-IR	−0.481	<0.05	−0.227	<0.05
LDL-C (mmol/L)	−0.215	0.051	−0.019	0.862
HDL-C (mmol/L)	−0.457	<0.05	0.054	0.702
Ln TG (mmol/L)	−0.374	<0.05	0.029	0.743
TC (mmol/L)	−0.350	<0.05	0.021	0.822
FFA (umol/L)	−0.385	<0.05	−0.272	<0.05

Significant *P* values (*P* < 0.05) are indicated in bold.

* Pearson correlation analysis was performed for irisin serum levels and the indicated parameters.

^†^ Partial correlation analysis after adjustment for SDS-BMI and age.

### lncRNA RP11-34D15.2 is downregulated in obese children and positively correlates with FNDC5/irisin expression

**Figure 1:**
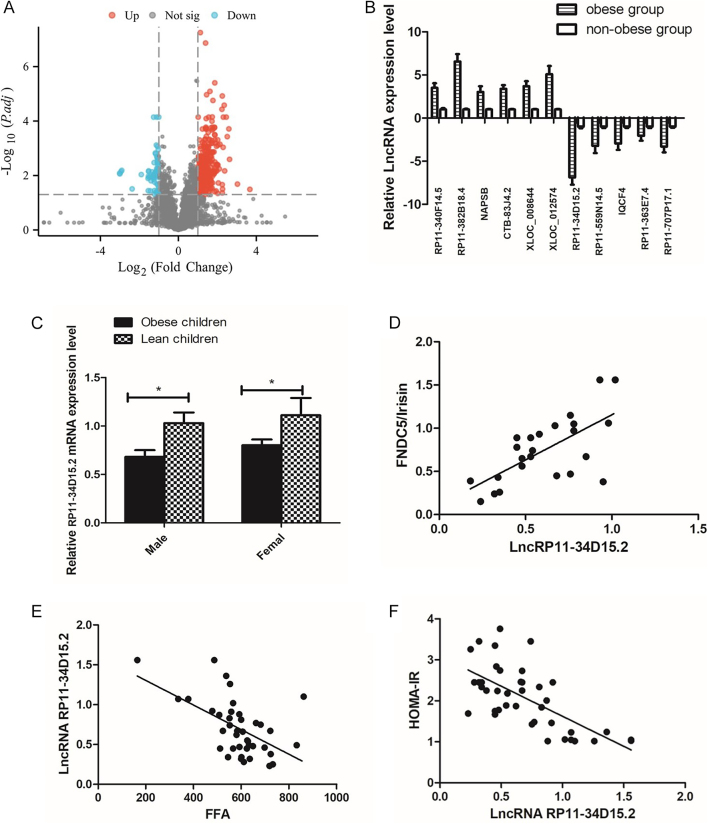
Expression profile of lncRNA RP11-34D15.2 in WAT of obese children. (A) Volcano plot identifies upregulated (red) and downregulated (blue) lncRNAs in WAT of obese (*n* = 3) versus non-obese (*n* = 3) children (fold change >2.0, *P* < 0.05). (B) Top ten differentially expressed lncRNAs ranked by log2 (fold change). (C) Quantitative PCR (qPCR) validation of lncRNA RP11-34D15.2 expression in WAT of obese and non-obese children (*P* < 0.01). (D) Positive correlation between lncRNA RP11-34D15.2 and FNDC5/irisin mRNA levels (*r* = 0.603, *P* < 0.05). (E and F) Negative correlations between lncRNA RP11-34D15.2 and serum FFAs (*r* = −0.556) or HOMA-IR (*r* = −0.470) (*P* < 0.05). Data: mean ± SD. **P < 0.01.

RNA microarray analysis of white adipose tissue (WAT) from three obese and three non-obese children identified a panel of differentially expressed lncRNAs (fold change >2.0, *P* < 0.05). The top ten upregulated and downregulated lncRNAs are visualized in the hierarchical clustering heatmap (Fig. 1A). Subsequent qRT-PCR validation in 40 obese and 40 normal-weight children confirmed lncRNA RP11-34D15.2 as the most significantly downregulated transcript in obese WAT (*P* < 0.01; Fig. 1B and C), with no gender-specific expression differences. Positive correlations were observed between lncRNA RP11-34D15.2 and FNDC5/irisin mRNA levels (*r* = 0.603, *P* < 0.05; Fig. 1D), while inverse relationships were detected with FFA (*r* = −0.556, *P* < 0.05) and HOMA-IR (*r* = −0.470, *P* < 0.05; Fig. 1E and F).

### FNDC5/irisin enhances mitochondrial fatty acid oxidation and thermogenesis in SW872 adipocytes

**Figure 2:**
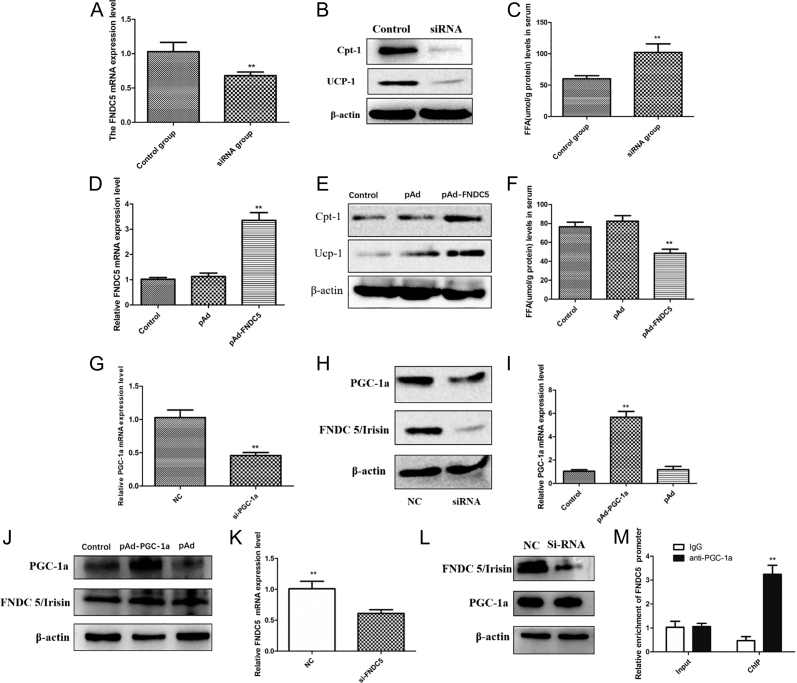
PGC-1α regulates FNDC5/irisin expression to enhance mitochondrial fatty acid oxidation and thermogenesis in SW872 adipocytes. (A) qRT-PCR analysis of FNDC5/irisin mRNA in FNDC5-knockdown cells. (B) Western blot showing reduced CPT-1 and UCP-1 protein levels post-FNDC5 silencing. (C) Elevated extracellular FFA in FNDC5-knockdown cells. (D) qRT-PCR analysis of FNDC5/irisin mRNA in FNDC5-overexpressing cells. (E) Western blot showing increased CPT-1 and UCP-1 protein levels post-FNDC5 overexpression. (F) Reduced extracellular FFA in FNDC5-overexpressing cells. (G) qRT-PCR analysis of PGC-1α mRNA in PGC-1α-knockdown cells. (H) Western blot showing decreased FNDC5 protein levels post-PGC-1α silencing. (I) qRT-PCR analysis of PGC-1α mRNA in PGC-1α-overexpressing cells. (J) Western blot showing increased FNDC5 protein levels post-PGC-1α overexpression. (K) qRT-PCR analysis of FNDC5/irisin mRNA in FNDC5-knockdown cells. (L) Impact of FNDC5 knockout on PGC-1α expression. (M) Co-immunoprecipitation (Co-IP) confirming physical interaction between PGC-1α and FNDC5/irisin. Data: mean ± SD. **P < 0.01.

Functional assays in SW872 preadipocytes demonstrated that FNDC5/irisin overexpression significantly upregulated mitochondrial markers CPT-1 and UCP-1 (*P* < 0.01; Fig. 2A, B, D, E), whereas FNDC5 knockdown suppressed their expression (*P* < 0.01). Concordantly, FFA levels in culture media were reduced in FNDC5-overexpressing cells (*P* < 0.01; Fig. 2C) and elevated in FNDC5-silenced cells (*P* < 0.01; Fig. 2F). These findings confirm FNDC5/irisin’s critical role in promoting mitochondrial fatty acid oxidation and thermogenic activity.

### PGC-1α regulates FNDC5/irisin expression in SW872 cells

PGC-1α knockdown significantly suppressed FNDC5/irisin protein and mRNA levels (*P* < 0.01; Fig. 2G and H), while its overexpression enhanced FNDC5/irisin expression (*P* < 0.01; Fig. 2I and J). A positive correlation between PGC-1α and FNDC5/irisin mRNA levels was observed in WAT of obese children (*r* = 0.347, *P* < 0.05). Co-immunoprecipitation assays confirmed direct physical interaction between PGC-1α and FNDC5/irisin (Fig. 2M), supporting a role for PGC-1 as a transcriptional coactivator of FNDC5.

### lncRNA RP11-34D15.2 acts as a ceRNA by sponging miR-223-5p to upregulate PGC-1α

**Figure 3:**
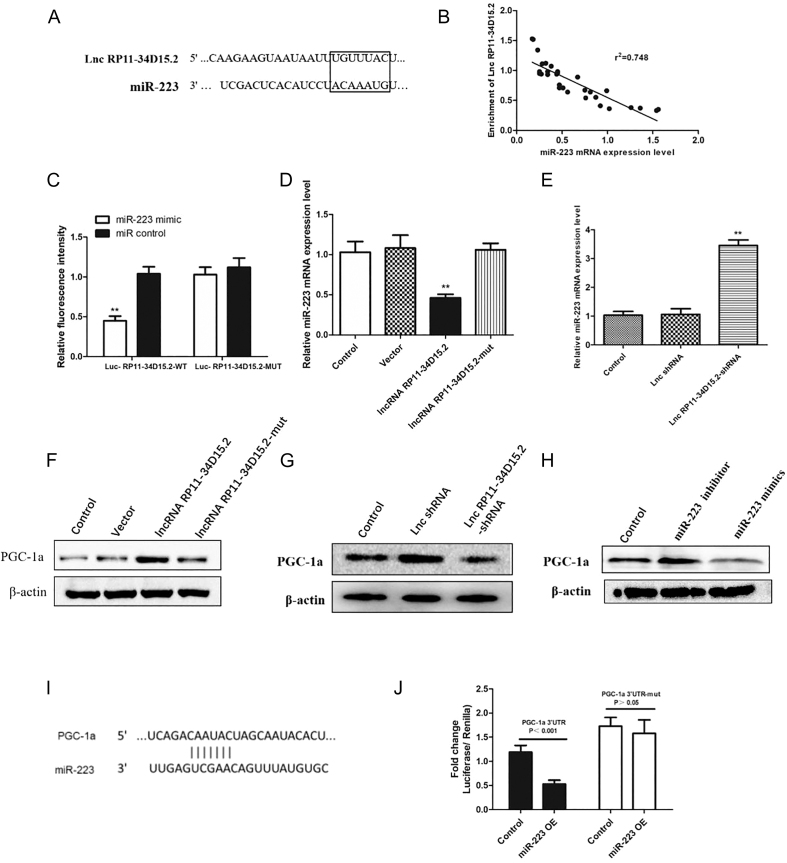
lncRNA RP11-34D15.2 functions as a ceRNA by sponging miR-223-5p to upregulate PGC-1α. (A) RNAhybrid-predicted binding sites between lncRNA RP11-34D15.2 and miR-223-5p. (B) Negative correlation between lncRNA RP11-34D15.2 and miR-223-5p levels in WAT (*r^2^* = 0.748, *P* < 0.01). (C) Luciferase reporter assay confirming direct interaction. (D and E) miR-223-5p levels in lncRNA RP11-34D15.2-overexpressing or -silenced SW872 cells. (F and G) Western blot analysis of PGC-1α protein levels in lncRNA RP11-34D15.2-modulated cells. (H) qRT-PCR analysis of PGC-1α mRNA in cells transfected with miR-223-5p mimics or inhibitor. (I) RNAhybrid-predicted binding sites between PGC-1α 3′-UTR and miR-223-5p. (J) Luciferase reporter assay validating miR-223-5p targeting of PGC-1α. Data: mean ± SD. **P < 0.01.

Bioinformatic analysis predicted a binding site between lncRNA RP11-34D15.2 and miR-223-5p, with complementary base pairing (7-nt sequence) and low minimum free energy (Fig. 3A). This interaction was corroborated by a strong negative correlation between lncRNA RP11-34D15.2 and miR-223-5p in obese WAT (*r^2^* = 0.748, *P* < 0.01; Fig. 3B). Luciferase reporter assays confirmed direct binding (*P* < 0.01; Fig. 3C). Functionally, lncRNA RP11-34D15.2 overexpression reduced miR-223-5p levels (*P* < 0.01; Fig. 3D), while its knockdown elevated miR-223-5p (*P* < 0.01; Fig. 3E). Furthermore, lncRNA RP11-34D15.2 positively regulated PGC-1α protein expression (Fig. 3F and G). miR-223-5p mimics suppressed PGC-1α expression (*P* < 0.01; Fig. 3H), whereas miR-223-5p inhibitors enhanced it (*P* < 0.01). Dual-luciferase assays validated binding of miR-223-5p to both lncRNA RP11-34D15.2 (*P* < 0.01; Fig. 3I) and PGC-1α 3′UTR (*P* < 0.01; Fig. 3J), establishing a competitive endogenous RNA (ceRNA) regulatory axis.

### *In vivo* validation of lncRNA RP11-34D15.2 knockdown in HFD-fed mice

**Figure 4:**
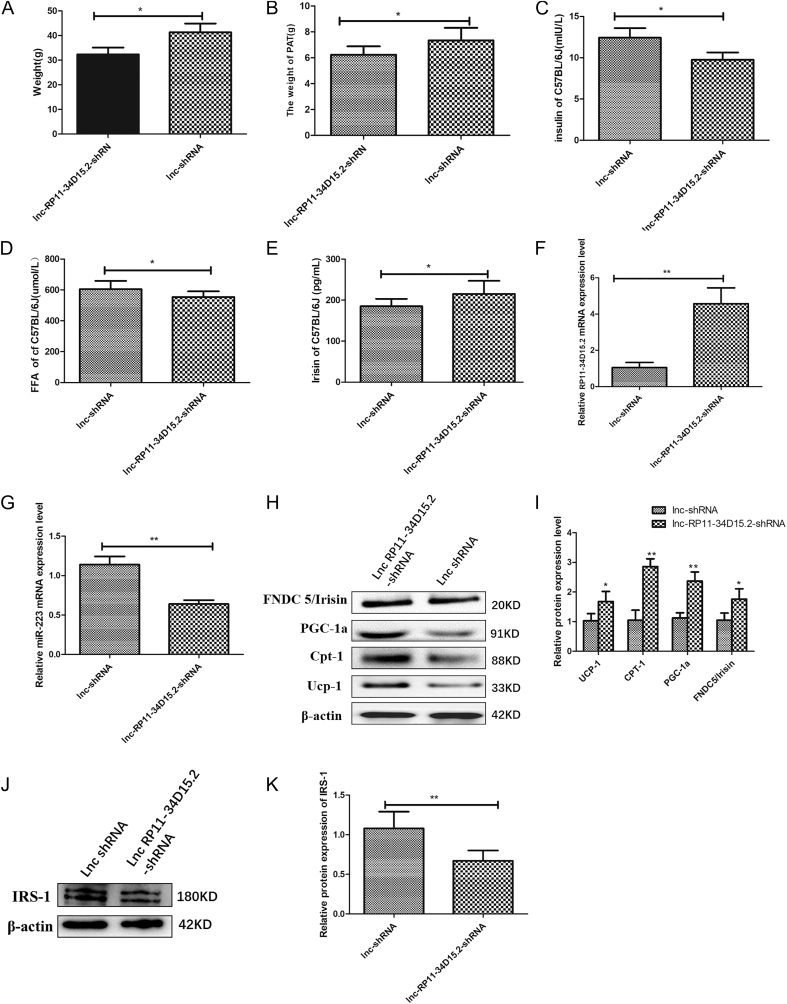
Metabolic and molecular effects of lncRNA RP11-34D15.2 knockdown in HFD-fed C57BL/6J mice. (A) Body weight. (B) PAT mass. (C and D) Serum insulin and FFA levels. (E) Serum irisin levels. (F and G) qRT-PCR analysis of lncRNA RP11-34D15.2 and miR-223-5p mRNA in PAT. (H and I) Western blot analysis of PGC-1α, FNDC5/irisin, CPT-1, and UCP-1 protein levels. (J and K) Western blot analysis of IRS-1 protein levels. FFA: free fatty acids. Data: mean ± SD. *P < 0.05; **: *P* < 0.01.

Body weight remained comparable between groups at baseline (*P* > 0.05) but diverged significantly 2 weeks post-treatment (*P* < 0.05; Fig. 4A). HFD-fed mice treated with lnc-RP11-34D15.2-shRNA exhibited significantly lower body weight (*P* < 0.05; Fig. 4A) and PAT mass (*P* < 0.05; Fig. 4B) compared to lnc-shRNA controls. The lnc-RP11-34D15.2-shRNA group demonstrated reduced serum FFA (*P* < 0.05; Fig. 4C) and insulin (*P* < 0.05; Fig. 4D), alongside elevated irisin levels (*P* < 0.05; Fig. 4E). qRT-PCR confirmed increased lncRNA RP11-34D15.2 (*P* < 0.01; Fig. 4F) and decreased miR-223-5p (*P* < 0.01; Fig. 4G) in PAT of knockdown mice. Western blotting revealed upregulated PGC-1α, FNDC5/irisin, CPT-1, and UCP-1 proteins in the lnc-RP11-34D15.2-shRNA group (*P* < 0.01 or *P* < 0.05; Fig. 4H and I). Conversely, IRS-1 expression was significantly lower in lnc-RP11-34D15.2-shRNA mice (*P* < 0.01; Fig. 4J and K).

## Discussion

This investigation establishes that lncRNA RP11-34D15.2 is significantly downregulated in the adipose tissue of obese pediatric patients, demonstrating positive correlation with FNDC5/irisin expression, while exhibiting inverse associations with both HOMA-IR indices and circulating FFA concentrations. Through mechanistic studies, we elucidate that lncRNA RP11-34D15.2 operates as a competitive endogenous RNA (ceRNA) by molecular sequestration of miR-223-5p, consequently alleviating its suppressive effect on the transcriptional coactivator PGC-1α. This regulatory cascade potentiates mitochondrial fatty acid oxidation and thermogenic capacity through FNDC5/irisin upregulation, ultimately mitigating pathological FFA accumulation and improving insulin sensitivity.

Skeletal muscle, now recognized as a principal endocrine organ, secretes myokines that exert pleiotropic effects on energy homeostasis and insulin sensitivity. Irisin, a PGC-1α-dependent myokine, has emerged as a critical modulator of mitochondrial metabolism, though its association with IR remains controversial in current literature. While investigations by Wu *et al.* (3) and Ulualan *et al.* (25) documented inverse correlations between irisin levels and obesity-associated IR, contradictory findings were reported by Sharan *et al.* (26). Our clinical data substantiate the former perspective, demonstrating: i) significantly diminished serum irisin concentrations in obese pediatric subjects, and ii) persistent negative correlations with fasting insulin, HOMA-IR, and FFA levels following adjustment for adiposity indices and age. These discordant observations may derive from population heterogeneity (e.g., age, ethnicity, metabolic status) or methodological variations in irisin quantification, underscoring the imperative for standardized assay protocols and longitudinal cohort studies to definitively establish irisin’s pathophysiological role in metabolic dysregulation.

The ceRNA hypothesis postulates that lncRNAs modulate gene expression networks by competitively binding microRNAs, thereby influencing miRNA-target interactions (27). Accumulating evidence implicates lncRNA-mediated ceRNA networks in adipogenic differentiation (28) and metabolic disease pathogenesis (29, 30), though their involvement in pediatric IR remains insufficiently characterized. Our study advances this paradigm by identifying lncRNA RP11-34D15.2 as a novel molecular sponge for miR-223-5p. The robust negative correlation between lncRNA RP11-34D15.2 and miR-223-5p in obese adipose tissue (*r*^2^ = 0.748), complemented by luciferase reporter validation of their direct interaction, establishes a mechanistic connection between this lncRNA and mitochondrial regulation via PGC-1α modulation.

While miR-223 has been predominantly studied in inflammatory and oncogenic contexts (31), emerging evidence suggests its dysregulation in obesity and T2DM pathophysiology (32). The documented PPARγ-mediated transcriptional control of miR-223 (33) implies the existence of a feedback loop between adipogenic signaling and mitochondrial metabolic regulation. Our experimental results demonstrate that miR-223-5p directly suppresses PGC-1α expression, a master regulator of mitochondrial biogenesis and β-oxidation. This suppression is counterbalanced by lncRNA RP11-34D15.2, which restores PGC-1α/FNDC5 signaling to augment expression of CPT-1 and UCP-1, both critical mediators of fatty acid catabolism and thermogenic capacity.

Several study limitations merit consideration:

- Sample size considerations: the moderate cohort size (*n* = 80) may constrain statistical power to detect subtle metabolic associations or subgroup differences.
- Mechanistic resolution: while luciferase assays confirmed miRNA-lncRNA interactions, advanced molecular techniques (e.g., RNA immunoprecipitation, CRISPR-Cas9 genome editing) would strengthen evidence for binding specificity and causal relationships.
- Pathway breadth: primary focus on CPT-1/UCP-1 may overlook additional mitochondrial targets; integrative omics approaches could delineate more comprehensive regulatory networks.
- Translational potential: preclinical findings require validation in human intervention trials to assess therapeutic applicability. In addition, the use of the SW872 cell line, which is a human liposarcoma cell line, presents a limitation, as its metabolic characteristics may not fully recapitulate those of primary human adipocytes or noncancerous preadipocyte cell lines.

Future investigations should: i) expand cohort diversity across ethnicities and metabolic phenotypes, ii) employ single-cell sequencing technologies to resolve adipose tissue subpopulation dynamics, and iii) explore combinatorial targeting strategies for lncRNA RP11-34D15.2 and miR-223-5p in metabolic intervention paradigms.

## Conclusions

In summary, this work delineates a novel lncRNA RP11-34D15.2/miR-223-5p/PGC-1α/FNDC5 regulatory axis that governs mitochondrial fatty acid metabolism and insulin sensitivity in pediatric obesity. Functioning as a ceRNA, lncRNA RP11-34D15.2 attenuates miR-223-5p-mediated repression of PGC-1α, thereby potentiating FNDC5/irisin-driven mitochondrial β-oxidation and thermogenic capacity. These findings not only elucidate a previously unrecognized molecular circuit in childhood IR but also nominate lncRNA RP11-34D15.2 as a promising candidate for both diagnostic biomarker development and targeted therapeutic strategies against obesity-associated metabolic dysfunction.

## Declaration of interest

The authors declare that there is no conflict of interest that could be perceived as prejudicing the impartiality of the work reported.

## Funding

This study was funded by the National Science Foundation of Chinahttps://doi.org/10.13039/501100001809 (No. 81803262), Basic Research of Natural Science in Shaanxi Province (No. 2020JQ-549), Institute Foundation Free Exploration Project-2020 (No. 2020YJ(ZYTS)014), and Research Fund of the Second Affiliated Hospital of Jiaotong University (No. YJ(ZD)201709).

## Author contribution statement

MG L and YS L contributed to the conception and design of the study. HG Z, GS, and BY L performed the experiments, collected and analyzed data. MG L wrote the manuscript. YF X and CY Y revised the manuscript. All authors reviewed and approved the final version of the manuscript.

## Data availability

The datasets analyzed during the current study are not publicly available due to personal privacy but are available from the corresponding author on reasonable request.

## Ethics approval and consent to participate

The current study was conducted in accordance with the Helsinki Declaration of the World Medical Association and approved by the Ethics Committee of the Second Affiliated Hospital of Xi’an Jiaotong University. Written informed consent was obtained from participating parents before enrollment. We confirm that all methods were carried out in accordance with relevant guidelines and regulations. All methods are reported in accordance with ARRIVE guidelines for the reporting of animal experiments. All the experimental protocols were approved by the Independent Ethical Committee of the Second Affiliated Hospital of Xi’an Jiaotong University.
